# Brain Networks Implicated in Seasonal Affective Disorder: A Neuroimaging PET Study of the Serotonin Transporter

**DOI:** 10.3389/fnins.2017.00614

**Published:** 2017-11-03

**Authors:** Martin Nørgaard, Melanie Ganz, Claus Svarer, Patrick M. Fisher, Nathan W. Churchill, Vincent Beliveau, Cheryl Grady, Stephen C. Strother, Gitte M. Knudsen

**Affiliations:** ^1^Neurobiology Research Unit, Copenhagen University Hospital Rigshospitalet, Copenhagen, Denmark; ^2^Faculty of Health Sciences, University of Copenhagen, Copenhagen, Denmark; ^3^Neuroscience Research Program, St. Michael's Hospital, Toronto, ON, Canada; ^4^Rotman Research Institute, Baycrest and Medical Biophysics, University of Toronto, Toronto, ON, Canada

**Keywords:** 5-HTTLPR, Seasonal Affective Disorder, Partial Least Squares, PET, [^11^C]DASB, neuroplasticity, reproducibility, prediction

## Abstract

**Background:** Seasonal Affective Disorder (SAD) is a subtype of Major Depressive Disorder characterized by seasonally occurring depression that often presents with atypical vegetative symptoms such as hypersomnia and carbohydrate craving. It has recently been shown that unlike healthy people, patients with SAD fail to globally downregulate their cerebral serotonin transporter (5-HTT) in winter, and that this effect seemed to be particularly pronounced in female S-carriers of the 5-HTTLPR genotype. The purpose of this study was to identify a 5-HTT brain network that accounts for the adaption to the environmental stressor of winter in females with the short 5-HTTLPR genotype, a specific subgroup previously reported to be at increased risk for developing SAD.

**Methods:** Nineteen females, either S' carriers (L_G_- and S-carriers) without SAD (*N* = 13, mean age 23.6 ± 3.2 year, range 19–28) or S' carriers with SAD (*N* = 6, mean age 23.7 ± 2.4, range 21–26) were PET-scanned with [^11^C]DASB during both summer and winter seasons (asymptomatic and symptomatic phase, 38 scans in total) in randomized order, defined as a 12-week interval centered on summer or winter solstice. We used a multivariate Partial Least Squares (PLS) approach with NPAIRS split-half cross-validation, to identify and map a whole-brain pattern of 5-HTT levels that distinguished the brains of females without SAD from females suffering from SAD.

**Results:** We identified a pattern of 5-HTT levels, distinguishing females with SAD from those without SAD; it included the right superior frontal gyrus, brainstem, globus pallidus (bilaterally) and the left hippocampus. Across seasons, female S' carriers without SAD showed nominally higher 5-HTT levels in these regions compared to female S' carriers with SAD, but the group difference was only significant in the winter. Female S' carriers with SAD, in turn, displayed robustly increased 5-HTT levels in the ventral striatum (bilaterally), right orbitofrontal cortex, middle frontal gyrus (bilaterally), extending to the left supramarginal gyrus, left precentral gyrus and left postcentral gyrus during winter compared to female S' carriers without SAD.

**Limitations:** The study is preliminary and limited by small sample size in the SAD group (*N* = 6).

**Conclusions:** These findings provide novel exploratory evidence for a wintertime state-dependent difference in 5-HTT levels that may leave SAD females with the short 5-HTTLPR genotype more vulnerable to persistent stressors like winter. The affected brain regions comprise a distributed set of areas responsive to emotion, voluntary, and planned movement, executive function, and memory. The preliminary findings provide additional insight into the neurobiological components through which the anatomical distribution of serotonergic discrepancies between individuals genetically predisposed to SAD, but with different phenotypic presentations during the environmental stressor of winter, may constitute a potential biomarker for resilience against developing SAD.

## Introduction

Seasonal Affective Disorder (SAD) is a condition characterized by the repeated occurrences of major depressive episodes during winter and with subsequent remission in the summer (Rosental et al., 1984; Johansson et al., 1999, 2003; Lam and Levitan, 2000; Kalbitzer et al., 2010). It is estimated that up to 6% of the American population is affected by SAD, and additionally 10–20% may suffer from sub-syndromal SAD also referred to as “winter blues” (Avery et al., 2001). Extensive research in humans supports a number of predisposing factors responsible for the development of neuropsychiatric disorders such as depression, including age (Buchert et al., 2006; Kalbitzer et al., 2009; Erritzoe et al., 2010; Melrose, 2015), sex (Erritzoe et al., 2010; Melrose, 2015), environment (Caspi et al., 2003; Kendler et al., 2005), personality traits (e.g., neuroticism) (Lesch et al., 1996; Greenberg et al., 2000), genotype (5-HTTLPR or BDNF val66met) (Hariri et al., 2003; Lang et al., 2009; MacQueen and Frodl, 2011; Wang et al., 2012; Fisher et al., 2015; McMahon et al., 2016) and serotonin transporter (5-HTT) levels (McMahon et al., 2016; Tyrer et al., 2016). The mechanism by which 5-HTT adjustments cause SAD, has mainly been attributed to a seasonal dysregulation of 5-HTTs (McMahon et al., 2016; Tyrer et al., 2016) which are localized on the presynaptic terminal, ensuring that extracellular serotonin is inactivated and recycled into the presynaptic neuron. The symptoms of SAD can successfully be alleviated by the use of antidepressant medication such as Selective Serotonin Reuptake Inhibitors (SSRIs) which block the reuptake of serotonin, and/or light therapy which increases blood serotonin. However, the areas in which these treatments target the brain, and maybe more importantly how the resilient brain differs from the diseased brain is largely unknown. Early cross-sectional Positron Emission Tomography (PET) studies using less 5-HTT selective radioligands have not shown any convincing season-dependent fluctuations in 5-HTT levels (Neumeister et al., 2000; Koskela et al., 2008; Cheng et al., 2011), whereas more recent PET studies using more selective radioligands and high-resolution PET scanners have reported higher 5-HTT levels in the winter compared to summer in the regions mesencephalon, thalamus, prefrontal cortex, cingulate cortex, caudate, and putamen (Buchert et al., 2006; Praschak-Rieder et al., 2008). A study by Kalbitzer et al. (2010) reported a significant interaction between gene and environment, with healthy S' carriers displaying larger seasonal 5-HTT fluctuations in the putamen and caudate compared to long allele (L_A_/L_A_) carriers, and with peak 5-HTT levels occurring in the wintertime (Kalbitzer et al., 2010). However, two PET studies of comparable size reported no seasonal fluctuations in 5-HTT levels, although only males were included in these latter studies (Murthy et al., 2010; Matheson et al., 2015). In a more recent study (McMahon et al., 2016) published the first [^11^C]DASB PET longitudinal study investigating whole-brain seasonal 5-HTT fluctuations in both patients with SAD and in healthy individuals (McMahon et al., 2016). They reported that a whole-brain seasonal change in 5-HTT predicted symptom severity in patients with SAD, and that the change was primarily driven by females with the short 5-HTTLPR genotype (S' carriers). Their findings have subsequently been corroborated by another lab (Tyrer et al., 2016).

Most prior studies investigating SAD have focused on defining specific neurobiological biomarkers associated with SAD or predictive of treatment (Avery et al., 2001; Best et al., 2011; Lanzenberg et al., 2012; Hahn et al., 2014; Yeh et al., 2015). To date, however, no reliable biomarkers of disease or treatment outcome in SAD have been identified. This may partly stem from a failure to proficiently take the sources of variation in the acquired data into consideration. In addition, shifting the focus from solely studying biomarkers in patients to instead investigate resilience, i.e., how people are able to withstand environmental stress, may constitute a novel and more promising strategy (King, 2016). The current study extends the work of McMahon et al. (2016) by directly investigating, in a longitudinal design, spatially distributed patterns of 5-HTT brain expression that discriminate female S' carriers without SAD from female S' carriers with SAD. We chose to investigate female S-carriers only, because intergroup variability can limit the interpretation of small studies and more importantly, because female S-carriers have been reported to be at increased risk for developing depression (Greenberg et al., 2000; Caspi et al., 2003; Kendler et al., 2005). Furthermore, it has been proposed that inconsistent reports of 5-HTT levels in SAD might be explained by the idea that differences in binding between regions are not independent (univariate), but rather occur on a network level (multivariate) (Spies et al., 2015). In this study, we used a multivariate data-driven approach to identify a spatially distributed pattern of 5-HTT levels that fluctuated coherently with group and season. Because SSRIs block the 5-HTT, we hypothesized that female S' carriers without SAD would show reduced 5-HTT levels in a specific set of brain regions, in response to the environmental stressor of winter, whereas female S' carriers with SAD would show elevated 5-HTT levels in the same regions (McMahon et al., 2016). We expected that such regions would include areas rich in 5-HT innervation such as the striatum, brainstem (raphe) and thalamus (Varnas et al., 2004), as well as regions involved in memory and learning, such as the hippocampus (MacQueen and Frodl, 2011). Additionally, one might expect to see engagement of the amygdala involved in the fear response, and frontal areas involved in emotional and cognitive control (Ochsner et al., 2002; Vincent et al., 2008).

## Materials and methods

### Participants

A total of 19 female subjects were recruited from a paired longitudinal study investigating seasonal 5-HTT fluctuations in SAD (see Table 1 for demographic information) (McMahon et al., 2016). Inclusion criteria included: (1) <45 years of age, (2) body mass index (BMI) of 19–28 kg/m^2^, (3) non-smokers, (4) stable diurnal cycle and (5) no past or present neurological/psychiatric disorders. All subjects had a psychometric assessment using the Major Depression Inventory (MDI) (Bech et al., 2001) and the Pittsburgh Sleep Quality Index global scores (PSQI) (Buysse et al., 1989), and these assessments were carried out both summer and winter concurrent with each PET scan. In addition, all subjects were evaluated for seasonal mood cycles using the Seasonal Pattern Assessment Questionnaire (SPAQ). If the SPAQ supported the nonexistence of season dependent mood cycles [Global Seasonality Score (GSS) ≤ 10] subjects were clinically classified as being healthy females displaying no psychopathology for SAD, whereas subjects with GSS >10 were classified as females with SAD. For additional information on menstrual cycle phase, exclusion of axis I or axis II disorders and exclusion for treatment effects we refer the reader to McMahon et al. (2016). All subjects were genotyped for the 5-HTTLPR polymorphism as described in McMahon et al. (2016), and only S' carriers (L_G_- and S-carriers) were included in the current study. All subjects were PET scanned with the radioligand [^11^C]DASB both summer and winter in a randomized counterbalanced fashion, with scanning occurring within 12-week intervals centered on summer or winter solstice. The final sample consisted of 13 female S'-carriers without SAD and 6 female S'-carriers with SAD that were all scanned twice (38 PET scans in total). All data used in this study have been reported in a previous PET study (McMahon et al., 2016). The protocol was approved by the Ethics Committee of Copenhagen (H-1-2010-085 with amendments and KF-01-2006-20 with amendment 21971/220225, H-1-2010-91 and H-2-2010-108). All subjects provided written informed consent prior to participation, in accordance with The Declaration of Helsinki II.

**Table 1:** Demographic information. s/w refers to summer/winter.

	**Non-SAD females**	**SAD females**	***P*-value**
N	13	6	
Age (Mean ± SD)	23.6 ± 3.2	23.7 ± 2.4	0.93
BMI (Mean ± SD)	22.8 ± 2.3	20.9 ± 1.7	0.09
MDI (s/w)	5.1/5.3	6/23.3	0.6/0.0001
PSQI global score (s/w)	3.0/3.4	4.8/6.2	0.08/0.01
Neuroticism (s)	81.8 ± 17.0	84.3 ± 16.6	0.9
GSS	4.3 ± 2.2	14.5 ± 2.1	<0.0001
Daylight minutes (s)	1,009 ± 36.7	1043 ± 9.9	0.05
Daylight minutes (w)	438 ± 14.3	475 ± 46.6	0.02

*Daylight minutes are a measure of the daylight minutes on the day of the PET scan. MDI, Major Depression Inventory; PSQI, Pittsburgh Sleep Quality Index global scores; GSS, Global Seasonality Score; P-values are from two-sample t-tests*.

### Magnetic resonance imaging acquisition

An anatomical 3D T1-weighted MP-RAGE sequence with matrix size = 256 × 256 × 192; voxel size = 1 × 1 × 1 mm; TR/TE/TI = 1550/3.04/800 ms; flip angle = 9° was acquired for all patients using a Siemens Magnetom Trio 3T MR scanner or a Siemens 3T Verio MR scanner. All single-subject MRI sequences were corrected for gradient nonlinearities according to Jovicich et al. (2006), in order to correct for spatial distortions and achieve optimal PET-MR co-registration. All of the acquired MR images were examined for structural abnormalities, as a criterion for subject inclusion.

### Positron emission tomography using [^11^C]DASB

All patients were scanned using a Siemens ECAT High-Resolution Research Tomography (HRRT) scanner with an approximate in-plane resolution of 2 mm, operating in 3D list-mode and with the highly selective radioligand [^11^C]DASB (Olesen et al., 2009). The imaging protocol consisted of a single-bed, 90 min transmission acquisition post injection of 592 ± 14 (mean ± SD) MBq, range 536–608 MBq, bolus into an elbow vein. PET data was reconstructed into 36 frames (6 × 10, 3 × 20, 6 × 30, 5 × 60, 5 × 120, 8 × 300, and 3 × 600 s) using a 3D-OSEM-PSF algorithm with TXTV based attenuation correction (image matrix, 256 × 256 × 207; voxel size, 1.22 × 1.22 × 1.22 mm) (Sureau et al., 2008; Keller et al., 2013). Preprocessing of the PET data at the subject-level included in-scan motion correction to a reference frame (frame 26) using AIR 5.2.5. Prior to alignment, each frame was smoothed using a 10 mm Gaussian 3D kernel and thresholded at the 20-percentile level. Alignment parameters were estimated for the smoothed PET frames 10–36 using AIR. Subsequently, the motion parameters were applied to the original PET data using a rigid transformation with a least squares cost-function, and resliced into a 4D motion corrected data set.

### Parametric images of 5-HTT binding

All MR scans were processed and analyzed using FreeSurfer (Fischl, 2012) (http://surfer.nmr.mgh.harvard.edu, version 5.3) and MATLAB R2013a (8.1.0.604) 64 bit (Mathworks Inc, MA). Single-subject PET time activity curve (TAC) images were initially summed over all frames in order to estimate a time-weighted 3D image for co-registration. The time-weighted PET image was aligned to the structural MRI using a rigid intra-subject multimodal registration utilizing a boundary-based cost function with 6 degrees of freedom (Greve and Fischl, 2009). The non-displaceable binding potential (BP_ND_) at the voxel-level was estimated in PETSurfer (Greve et al., 2014) using the Multilinear Reference Tissue Model 2 (MRTM2) (Kim et al., 2006) with the cerebellum as a reference region, and thalamus, caudate, putamen and pallidum as high-binding regions for estimation of k_2_' (min^−1^). k_2_' is the clearance rate constant from reference region to plasma. The TAC image was normalized to MNI152 space using the structural MRI and the CVS registration algorithm from FreeSurfer, as this step has been shown to be particularly sensitive to both cortical and subcortical alignment (Zöllei et al., 2010). Voxel-wise group analysis was performed in the MNI152 space (Beliveau et al., 2015). All TAC images were smoothed with a Gaussian 3D filter using a full width half maximum (FWHM) of 5 mm. Kinetic modeling using MRTM2 was subsequently applied in order to estimate parametric images of 5-HTT binding (BP_ND_ images). To limit the contribution of low-binding white matter and cerebrospinal fluid voxels to the subsequent statistical analyses, BP_ND_ images (voxel-size: 1 × 1 × 1 mm) were masked to include only voxels with a gray matter probability >0.1 and cerebrospinal fluid probability <0.3 based on SPM8 probability templates (Fisher et al., 2015). The Partial Least Squares (PLS) analysis uses information from all voxels at the same time, so this masking was performed to ensure that ventricle voxels and voxels with low probability of being gray-matter was excluded. The mask was validated visually, and was used similarly in Fisher et al. (2015).

### Partial least squares analysis

The multivariate analysis was performed using a contrast-based PLS model as described in Churchill et al. (2013). PLS is a data-driven supervised form of Principal Component Analysis (PCA) that seeks to find shared information between two data matrices, identifying brain patterns in neuroimaging data matrix **X** that covary with contrast or experimental conditions in data matrix **Y** (McIntosh et al., 1996; Krishnan et al., 2011). Individual subject brain images were stored as row vectors in matrix **X** (scans × voxels). Rows of data matrix **Y** included two factors associated with each scan; seasonal condition (summer and winter) and group status (female S' carriers without SAD, and female S' carriers with SAD). This corresponds to a (scans × 4) design matrix coding for **Y**. Prior to running the PLS analysis, both **X** and **Y** were standardized columnwise (mean subtracted and normalized by the standard deviation), and the cross-product matrix **E** between **X** and **Y** (**E** = **Y**^T^**X**) was decomposed using singular value decomposition (SVD) to extract voxel-wise brain patterns that explained the greatest covariance across the experimental conditions stored in **Y**. The SVD decomposes **E** into three matrices, namely **E** = **UΔV**^T^. The left singular vectors **U** represent the contrast that best characterize **E**, whereas the right singular vectors **V** represent the voxels that best characterize **E**. In the PLS literature the singular vectors are commonly referred to as saliences. The singular values belonging to each singular vector pair is stored in Δ. The PLS analysis was carried out in the NPAIRS split-half cross-validation framework, which provides empirical estimates of the reliability of the 5-HTT spatial brain patterns and unbiased estimates of the strength of brain-contrast correlations. The analysis was performed by randomly assigning the subjects 1,000 times to split-half matrices **X**_1_ and **X**_2_, and experimental vectors **Y**_1_ and **Y**_2_. We used balanced stratified group and season subsampling in the splitting process (i.e., matched sampling), ensuring that each split-half represented both groups and seasons. For each split-half pair *J*, we estimated the projected latent variable (LV) brain pattern that explained the most variance **E**_i_ = ${\text{Y}}_{\text{i}}^{\text{T}}$**X**_i_ for *i* = 1, 2. The correlation between the design matrix coding and the corresponding expression of the latent brain pattern **E**_i_ for each split-half training set defined as *r*_(*i,train*)_ = ρ(**Y**_i_, **X**_i_${\text{E}}_{\text{i}}^{\text{T}}$) for *i* = 1, 2, where ρ is the Pearson linear correlation. In addition, we obtained an independent test measure of the experimental prediction power of each **E**_i_ by estimating *r*_(*i,test*)_ = ρ(**Y**_*j*≠*i*_, **X**_*j*≠*i*_${\text{E}}_{\text{i}}^{\text{T}}$) for *i* and *j* = 1, 2. The reproducibility of the two split-half brain patterns was measured as the correlation of all paired LVs, *r*_*spatial*_ = ρ(**E**_1_, **E**_2_), which measures the stability of the latent brain patterns across independent split-halves. The PLS analysis was executed on an adaptive optimized PC-subspace maximizing the contrast-correlation and the spatial reproducibility, as described in Churchill et al. (2013, 2014). A mathematical derivation and explanation of this procedure can be found in the Supplementary Material. The PC-subspace was varied from 1 to *k* PCs with *k* = *19* (total number of scans in a split-half), and its performance was defined as the Euclidean distance *D* from reproducibility = 1 and prediction = 1. This is consequently the PC-subspace that simultaneously maximizes the mean predictive correlation and mean spatial reproducibility of a given LV, and can mathematically be expressed as D(k) = min (1– r(k)_spatial_)^2^+(1– r(k)_test_)^2^. In a PLS analysis, each component pair produces LVs of maximum covariance between **X** and **Y**. Furthermore, as each LV explains a covarying pattern across the entire brain, the significance of the whole-brain pattern is determined, rather than estimating an independent *P*-value for every single voxel (McIntosh et al., 1996). The significance of each LV was determined across 1,000 split-half resamples using the empirical distribution of *r*_*test*_ and *r*_*spatial*_ and testing relative to 0. This provides a *P*-value for the reproducibility and prediction of the whole-brain brain pattern. LVs with a *P* < 0.05 were considered statistically significant, and were kept for further analysis. In PLS, each brain voxel has a weight (salience), which is directly related to the covariance of its activity with the contrast on each LV. To determine the robustness of these saliences we estimated unbiased standard errors (SE) for each voxel using NPAIRS split-half cross-validation with 1,000 splits. All voxels where the ratio of a salience to the SE for that voxel (Z-score_split_) exceeded ± 2.8 (*P* ≤ 0.005) were considered to robustly contribute to the brain pattern. These scores are analogous to Z-scores, and clusters of voxels mapping onto the construct defined by each LV were identified using a cluster extent threshold of 640 voxels, as described in Grady et al. (2013) and Fisher et al. (2015). To summarize our PLS analysis, we estimated “brain scores” of each participant's expression of each LV brain pattern by multiplying each voxel's salience by the normalized 5-HTT binding in the corresponding voxel, and summed over all brain voxels for all subjects. This can mathematically be expressed as, **brain score = XV**. The estimated brain scores are similar to the factor scores from a principal component analysis. The brain scores for each subject per condition were subsequently mean-centered by the grand-mean across conditions (i.e., group and season), and a 95% confidence interval (CI) was built around the mean brain score for all conditions using bootstrap resampling on the brain scores within each group/condition (Grady et al., 2013; Fisher et al., 2015). Significant differences in mean brain scores between groups and conditions were established by paired and two-sample *t*-tests, and corrected for multiple comparisons using Bonferroni correction. To evaluate the effect of PC-subspace optimization, we also compared the optimization with the mean predictive correlation and mean spatial reproducibility estimated directly on **X**. In addition, we compared the covariance explained for a given retained LV both with and without optimization. To further evaluate the robustness of the results across different choices of *k*, and to resolve any possible bias issues, we also correlated the salience brain pattern for *k* = min(D) to the remaining *k*'s including the salience brain pattern without PC-subspace optimization. All voxel coordinates reported are in MNI space.

## Results

**Figure 1:**
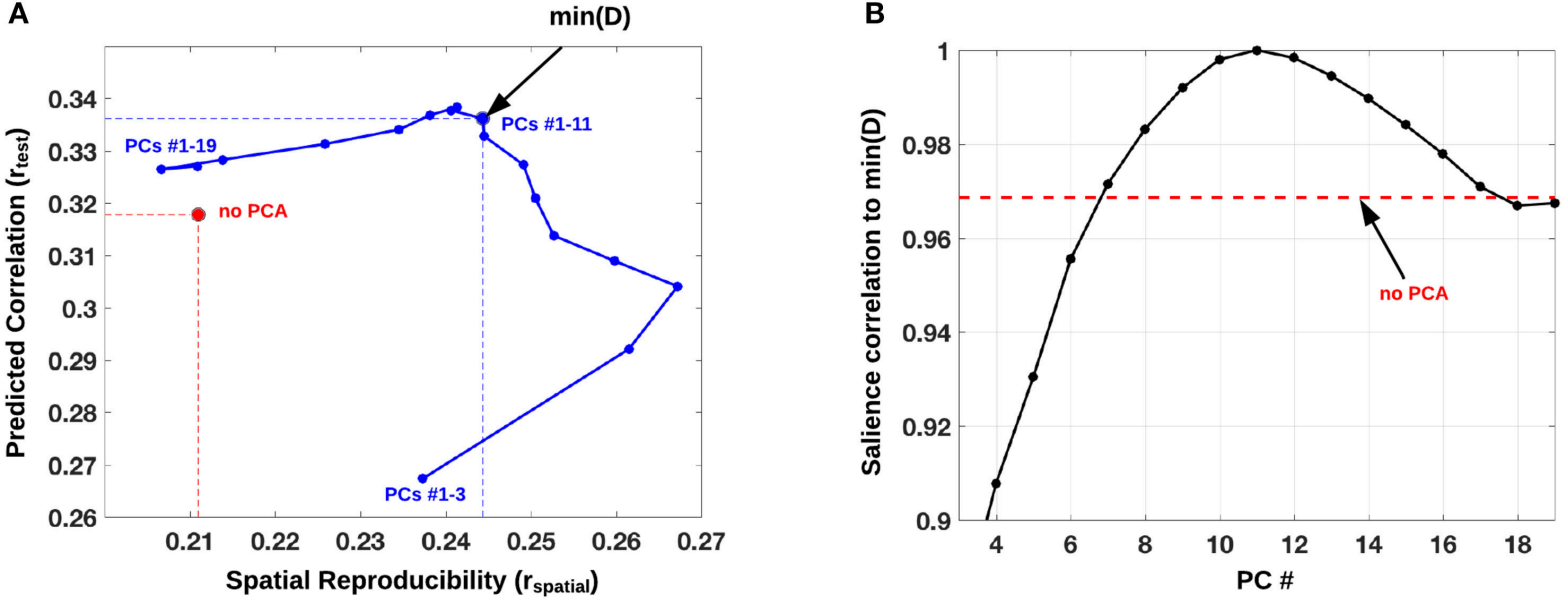
**(A)** PLS analysis performed on an adaptive optimized PCA subspace with mean predicted contrast-correlation of the significant LV plotted against the mean spatial reproducibility (blue). This PCA subspace was varied from 1 to *k* PCs with *k* = 19 (total number of scans in a split-half), and its performance was defined as the distance *D* from *r*_*spatial*_ = 1 and *r*_*test*_ = 1. A subspace of PCs 1–6 minimized *D*, which is displayed as a blue dot with a black circle. As a reference we also plot the mean (*r*_*spatial*_*, r*_*test*_*)* point, directly estimated from matrix **X** (red circle). **(B)** Correlation of brain pattern (salience) to min(D, *k* = 11) across different choices of *k* (PC #). The red dotted line indicates the correlation between the salience brain pattern for min(D, *k* = 11) and the salience brain pattern without PC-subspace optimization.

Demographic information and evaluation (two-sample *t*-tests) are listed in Table 1. The PLS analysis identified one LV with significant contrast prediction and spatial pattern reproducibility (P_test_ = 0.035 and P_spatial_ = 0.029) which was therefore retained for further analysis. This LV explained 70% of the total covariance across the two scanning sessions. A plot of the predicted brain-contrast correlation (*r*_*test*_) and the brain-pattern reproducibility (*r*_*spatial*_) of the LV brain map for PLS performed on an adaptive optimized PC subspace is presented in Figure 1A. From the plot in Figure 1A we identified the PC subspace (*k* = 11) that simultaneously maximized the mean predictive correlation and mean spatial reproducibility of the retained LV. In this PC-subspace we were able to obtain a 16% relative increase in mean spatial reproducibility compared to the reference mean estimate directly estimated from **X** (Figure 1A), and the relative mean predictive correlation was 6% higher than the reference estimate (no PCA). The reference LV estimated directly from **X** was only significant for *r*_*test*_ (P_test_ = 0.04) but not for *r*_*spatial*_ (P_spatial_ = 0.071), and the total covariance explained by this LV was instead 63%, corresponding to a relative decrease of 11% compared to the optimized analysis.

**Figure 2:**
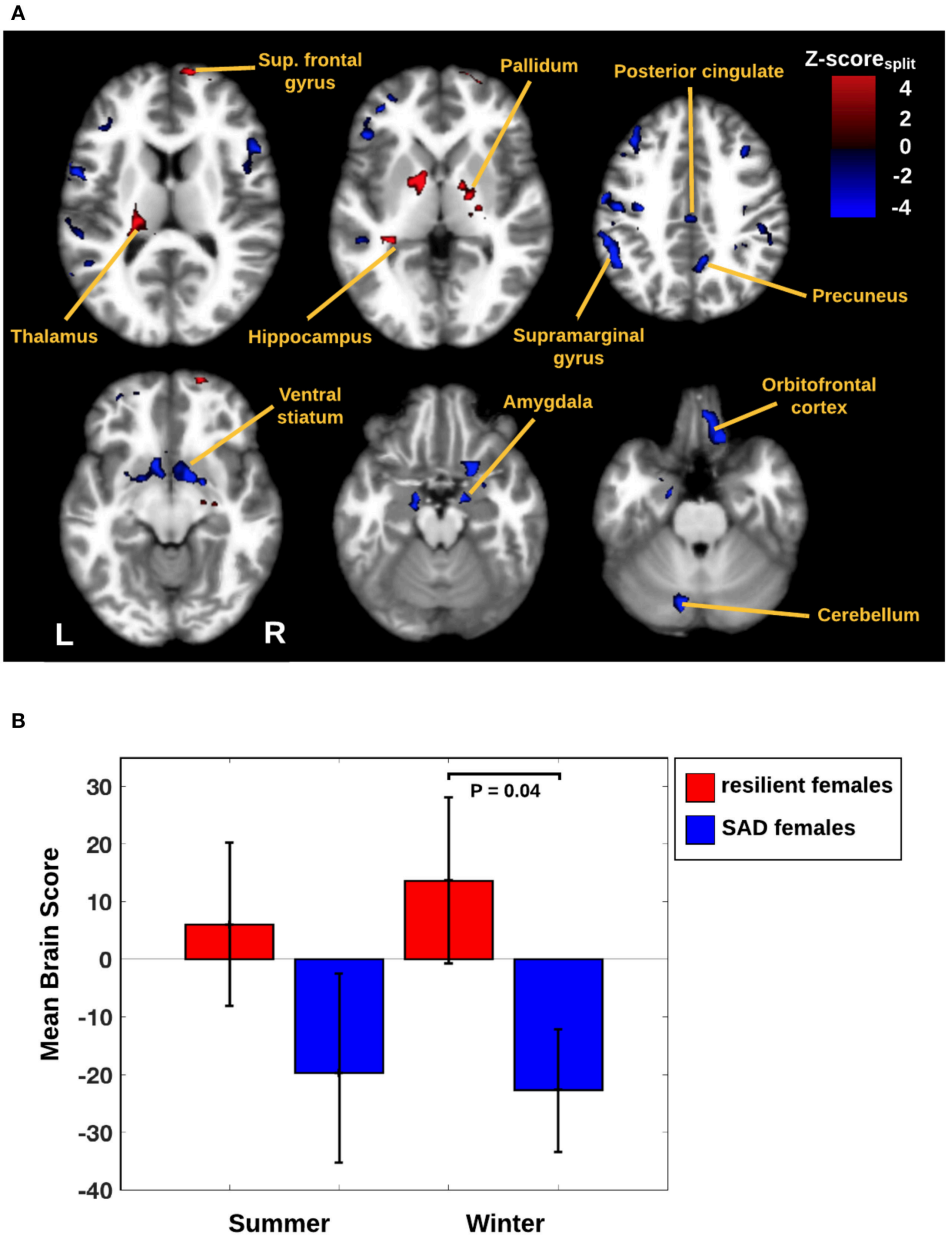
**(A)** Brain network predicted by the PLS analysis. The image is thresholded at |*Z*-score_split_| > 2.8 (*P* ≤ 0.005) and with cluster extent threshold > 640 voxels. Warm colors represent an increased 5-HTT response for female S' carriers without SAD compared to female S' carriers with SAD. Cool colors represent an increased 5-HTT response in female S' carriers with SAD compared to female S' carriers without SAD. Scale reflects Z-score_split_ units. Slices are in MNI coordinates. **(B)** Mean brain scores for seasonal conditions by either female S' carriers without SAD or female S' carriers with SAD. A mean brain score of zero indicates the mean brain response across all conditions. Error bars indicate 95% confidence interval for the brain scores.

**Figure 3:**
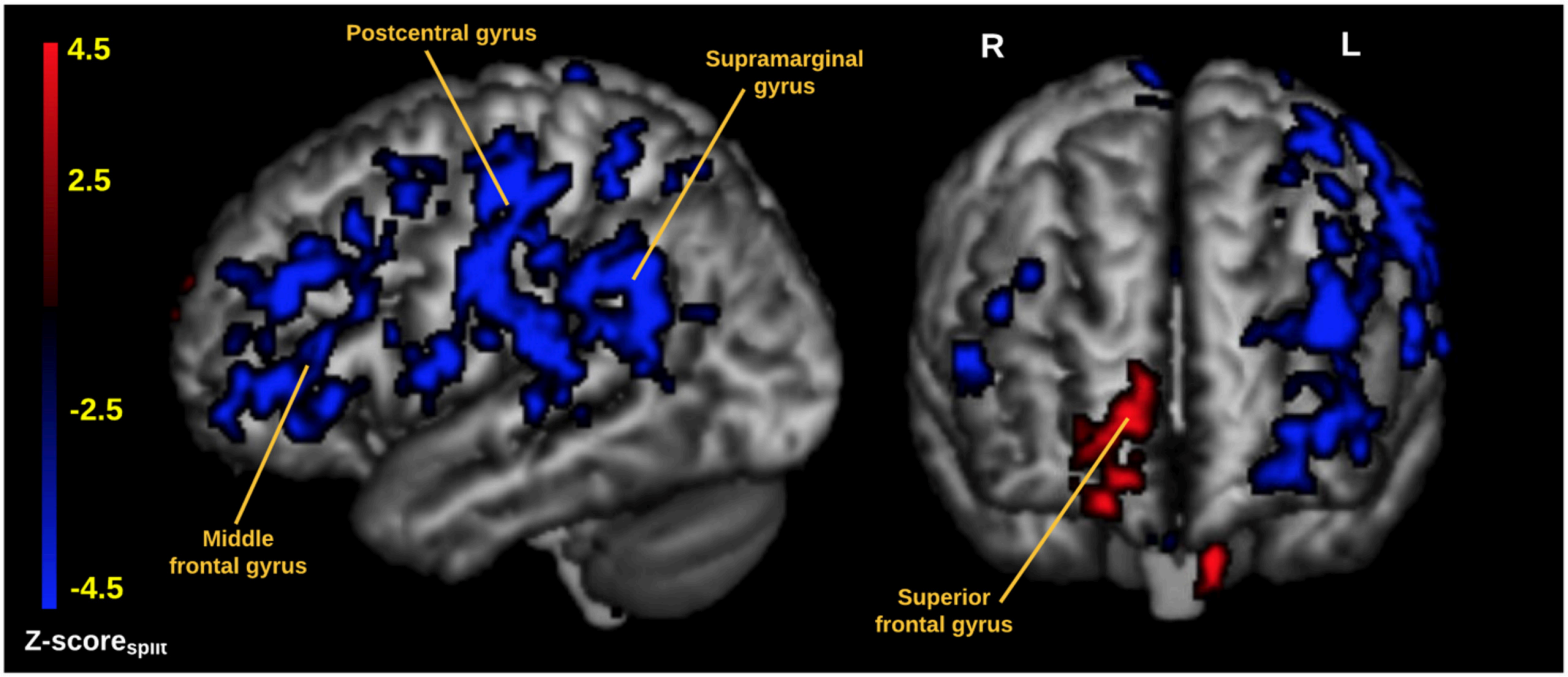
Cortical rendering of the 5-HTT brain network predicted by the PLS analysis. The image is thresholded at |Z-score_split_| > 2.8 (*P* ≤ 0.005) and with cluster extent threshold > 640 voxels. Red areas represent an increased 5-HTT response for female S' carriers without SAD compared to female S' carriers with SAD. Blue areas represent an increased 5-HTT response in female S' carriers with SAD compared to female S' carriers without SAD. The data is visualized using mricron (https://www.nitrc.org/projects/mricron).

In Figure 1B we display the robustness of the identified LV brain pattern (*k* = 11) given by its correlation with brain LV patterns across different choices of *k*. The identified LV brain pattern seems to be relatively robust (correlation >0.9) over various choices of *k*, suggesting that any possible bias issues are resolved with respect to optimizing the hyper parameter *k*. The voxel saliences identified a pattern of 5-HTT levels in the brain (Figure 2A) dissociating female S' carriers without SAD from female S' carriers with SAD, across seasonal conditions (Figure 2B). This pattern included a distributed set of brain regions, such as the brainstem, globus pallidus (bilaterally), ventral striatum (bilaterally), amygdala (bilaterally) and the left hippocampus. The pattern also included the right orbitofrontal cortex (bilaterally), middle frontal gyrus (bilaterally), right superior frontal gyrus, left supramarginal gyrus, left precentral gyrus and left postcentral gyrus (Table 2 and Figure 2A). This particular set of brain regions showed a selective 5-HTT brain pattern across season with female S' carriers without SAD expressing significantly higher 5-HTT binding in the winter (95% CI: 13.61 [−0.79; 28.07]) in the subcortical regions globus pallidus (bilaterally), left hippocampus, brainstem and in the right superior frontal gyrus, compared to female S' carriers with SAD (95% CI: −22.64 [−33.5; −12.26]). This is indicated by the warm colors in Figures 2A, 3. In contrast, female S' carriers with SAD showed significantly increased 5-HTT levels compared to females without SAD in the wintertime, with greatest effects in the ventral striatum (bilaterally) and amygdala (bilaterally), extending toward the right orbitofrontal cortex, middle frontal gyrus (bilaterally), and the left somatosensory cortex mostly covering the supramarginal gyrus, precentral gyrus, and postcentral gyrus (indicated by cold colors in Figures 2A, 3). We did not observe any significant difference in brain score between females without SAD (95% CI: 5.96 [−8.14; 20.16]) and SAD females (95% CI: −19.75 [−35.37; −2.58]) in the summertime. Neither did we observe any significant difference in brain scores when comparing SAD females in the wintertime (95% CI: −22.64 [−33.5; −12.26]) with both females without SAD in the summertime (95% CI: 5.81 [−9.18; 21.75]) and SAD females in the summertime (95% CI: −18.97 [−30.65; −6.1]). A table of the exact differences estimated by paired and two-sample *t*-tests and subsequently Bonferroni corrected can be found in Table 3 These results suggest that female S' carriers without SAD compared to females with SAD have *elevated 5-HTT levels* in a distributed set of regions spanning the brainstem, left hippocampus, globus pallidus (bilaterally) and right superior frontal gyrus in the wintertime, whereas they have *reduced 5-HTT levels* in the ventral striatum (bilaterally), amygdala (bilaterally), right orbitofrontal cortex, middle frontal gyrus (bilaterally), and left supramarginal gyrus, left precentral gyrus, and left postcentral gyrus.

**Table 2:** Local maxima of robust brain patterns identified from the PLS analysis and the corresponding coordinates in MNI-space.

**Location**	**Size [mm^3^]**	**X**	**Y**	**Z**	**Z-score_split_**
**LEFT HEMISPHERE**					
L hippocampus/thalamus	1,546	32	−35	0	5.31
L ventral striatum/amygd	2,318	−15	3	−7	−7.00
L brainstem	942	7	−48	−46	5.51
L pallidum	1,024	12	1	−1	5.20
L parstriangularis	3,931	36	45	1	−4.61
L middle front. gyrus	2,656	39	41	26	−5.4
L supramarginal gyrus	17,224	65	−23	23	−8.2
L cerebellum	765	12	−74	−16	−7.92
**RIGHT HEMISPHERE**					
R ventral striatum/amygd	1,818	15	−3	−19	−6.69
R med. orbifrontal cortex	1,064	−14	26	−25	−6.31
R paracentral	855	−5	−32	−66	−5.55
R post. cingulate	996	−4	−28	27	−5.53
R parsopercularis	2,934	−55	19	12	−5.19
R supramarginal gyrus	1,683	−53	−18	29	−4.96
R pallidum	1,390	−13	−4	−3	4.90
R sup. frontal gyrus	1,031	−14	65	10	4.89
R precentral gyrus	1,111	−23	−7	44	−4.30
R precuneus	673	−9	−48	38	−4.09

**Table 3:** Differences in brain scores within groups (paired *t*-tests), between groups (two-sample *t*-test), and across group and conditions (two-sample *t*-tests).

**Contrast (brain scores)**	***P*-value (uncorrected)**	***P*-value (Bonferroni)**
HC_summer_ vs. HC_winter_	0.0617	0.37
SAD_summer_ vs. SAD_winter_	0.852	5.11
HC_summer_ vs. SAD_summer_	0.068	0.41
HC_summer_ vs. SAD_winter_	0.033	0.198
HC_winter_ vs. SAD_summer_	0.0173	0.104
HC_winter_ vs. SAD_winter_	0.0068	0.04^*^

The obtained P-values are Bonferroni corrected to account for the number of statistical tests. Significance is given by

* *P < 0.05 (bold) after correction for multiple comparisons via Bonferroni correction*.

## Discussion

Our PLS analysis revealed that, relative to SAD female S' carriers, female S' carriers without SAD showed a wintertime state-dependent increase in 5-HTT levels in the brainstem, extending to the left hippocampus, globus pallidus (bilaterally) and right superior frontal gyrus. In addition, female S' carriers without SAD showed lower 5-HTT levels in the ventral striatum (bilaterally), amygdala (bilaterally), right orbitofrontal cortex, middle frontal gyrus (bilaterally), left supramarginal gyrus, left precentral gyrus, and left postcentral gyrus in the wintertime compared to female S' carriers with SAD. Our data builds on a subset of data from McMahon et al. (2016) who investigated whole brain 5-HTT levels as a primary outcome measure, arguing that 5-HTT binding is highly correlated across brain regions, because seasonal changes have been reported to affect various brain regions. We here extend the study of McMahon et al. (2016) to include a more detailed analysis of the specific regional changes that occur in female S' carriers without SAD vs. female S' carriers with SAD. More specifically, a voxel-based analysis could reveal both up- or down-regulation (positive and negative voxel saliences) that would not be disclosed by an averaged whole-brain analysis. If so, a whole brain 5-HTT measure would be too crude a simplification to disentangle the neurobiological network components contributing to the dissociation between female S' carriers without SAD and female S' carriers with SAD. To our knowledge this is the first longitudinal PET study that aims to identify a 5-HTT brain network that dissociates the healthy brain from the SAD brain in females with the short 5-HTTLPR polymorphism. We chose to investigate female S-carriers only, because intergroup variability can limit the interpretation of small studies and more importantly, because female S-carriers have been reported to be at increased risk for developing SAD (Greenberg et al., 2000; Caspi et al., 2003; Kendler et al., 2005). The role of the 5-HTT in non-seasonal depression has been extensively discussed in the literature with inconsistent results (Gryglewski et al., 2014), thus suggesting that subject/symptom heterogeneity may be of paramount importance for stratifying patients into clinical subgroups. The role of the 5-HTT is to recycle serotonin back into the presynaptic neuron, thereby inactivating synaptic serotonin function (Kalbitzer et al., 2010; McMahon et al., 2016). However, the expression and function of 5-HTT in the re-uptake of serotonin is dynamically controlled, and there exist two hypotheses related to the neuroplasticity or potentiation of 5-HTT; the first hypothesis (H_1_) supports that 5-HT might control its own reuptake, since elevated levels of 5-HT have been proven to limit the internalization of 5-HTT, consequently suggesting that low 5-HT concentrations result in a 5-HTT downregulation (Milak et al., 2005). The second hypothesis (H_2_) is related to the action of SSRIs which are thought to increase the synaptic serotonin level (Michalak et al., 2007; Westrin and Lam, 2007; Muller and Jacobs, 2010). SSRIs block the reuptake of serotonin, thereby increasing extracellular serotonin concentration, and reducing 5-HTT expression on the presynaptic cell membrane (Horschitz et al., 2001). The latter hypothesis is of particular interest in a pharmacological sense, as 30–50% of patients do not respond to SSRI treatment and the reason is still unknown (Nakajima et al., 2011). This means, however, that we are left with the question whether (1) a biological variation of 5-HTT induces changes in 5-HT, or (2) primary changes in 5-HT induce secondary changes in 5-HTT. Numerous previous studies investigating SAD have used a priori knowledge on Major Depressive Disorder related brain regions (i.e., amygdala, mPFC and anterior cingulate cortex), to infer whether these regions contribute to a 5-HTT modulation when exposed to the environmental stressor of winter (Praschak-Rieder et al., 2008; Tyrer et al., 2016). Anterior cingulate cortex did not significantly contribute to the pattern identified here, despite previous studies linking this region to SAD (Praschak-Rieder et al., 2008; Tyrer et al., 2016). In addition, we did not observe any significant differences in the summertime, and are therefore not able to predict the extent to which any of these subjects will develop SAD. This suggests that the serotonin transporter, based on these admittedly limited data, is *not* a biomarker to predict resilience vs. vulnerability in SAD. However, our results do support a tendency of increased 5-HTT levels in the ventral striatum, orbifrontal cortex, amygdala and largely lateralized areas of the left frontal and parietal cortices in the SAD group both at summer and winter. Depression has been associated with inter-hemispheric imbalances, suggesting the existence of a hyperactive right hemisphere for processing of negative emotions and a hypoactive left hemisphere for the processing of rewarding experiences (Hecht, 2010). While we observed only minor differences between females without SAD and females with SAD in 5-HTT levels in the right hemisphere, we instead observed increased 5-HTT levels in the left hemisphere in the SAD group, suggesting reduced synaptic serotonin levels and possibly reduced processing of rewarding experiences. The ventral striatum plays a key role for reward processing (Lanzenberg et al., 2012; Hahn et al., 2014), and is activated in response to stimuli such as sexual excitement and monetary rewards. Dysfunction of the ventral striatum has commonly been demonstrated in MDD (Hahn et al., 2014; Spies et al., 2015), but with less success in the SAD literature (Murthy et al., 2010; Matheson et al., 2015). Furthermore, the ventral striatum is a major target for deep brain stimulation (Lanzenberg et al., 2012), and light therapy works on the ventral striatum by reducing synaptic serotonin levels (Tyrer et al., 2016). Light therapy has also been shown to modulate an amygdala-prefrontal cortex corticolimbic circuit as measured with fMRI, and 5-HTTLPR genotype significantly moderated bright light intervention effects (Fisher et al., 2014). While our results mainly support an increase in 5-HTT levels in female S' carriers with SAD compared to female S' carriers without SAD, we observed increased levels of 5-HTT in the right middle frontal gyrus, left thalamus, bilateral pallidum and left hippocampus in the group without SAD. These regions are also part of the reward circuit including the amygdala and ventral striatum. However, while our results not only support a network of brain areas contributing to SAD, they may support a model for resilience with increased 5-HTT in the right middle frontal gyrus, left thalamus, bilateral pallidum and left hippocampus and lower 5-HTT in the amygdala and ventral striatum in order to sustain resilient to SAD. This supports the H_1_ hypothesis in the regions of increased 5-HTT in the phenotype without SAD, with limited internalization of 5-HTT and elevated levels of 5-HT (Milak et al., 2005). The observed interactions between up- and downregulations of 5-HTT are thus largely unknown, and future studies are warranted to explain the circuits and how they may impact other serotonin receptors (King, 2016). In addition, the presented results have implications for a necessary screening of female S' carriers with SAD in future studies, as subjects may enter a study as healthy participant in the summertime, but will have similar 5-HTT distribution in both their asymptomatic and symptomatic phase. This might result in a recruitment bias.

The effects of 5-HTTLPR on neural responses to emotionally salient stimuli have also been widely studied, mainly focusing on amygdala-prefrontal interactions (Holmes, 2008; Fisher and Hariri, 2012). In a recent study by Fisher et al., 2015, it was specifically demonstrated that S' carriers showed increased fMRI BOLD response to fearful faces compared to L_A_L_A_ carriers, and that this effect was mostly pronounced in a distributed set of brain regions including the amygdala (bilaterally), hippocampus (bilaterally), left middle frontal gyrus and superior frontal gyrus (bilaterally) (Fisher et al., 2015). Our results indicated that SAD female S' carriers have elevated 5-HTT levels in the wintertime in both the amygdala and middle frontal gyrus compared to female S' carriers without SAD. That is, these two regions behave similarly to emotionally salient stimuli, i.e., when confronted with an angry face paradigm. By contrast, we here observed *reduced* hippocampal 5-HTT levels in SAD females compared to females without SAD, suggesting that when it comes to hippocampal 5-HTT, these two phenotypes respond differently to the environmental stressor of winter. PLS is conceptually an advantageous technique compared to univariate approaches in cases where we have an a priori hypothesis that changes are not regional, but rather occur on a network level at multiple levels of brain function. Furthermore, PLS encompasses intriguing aspects, since the method is data-driven, and because its multivariate nature allows for alternative significance tests that do not require correction for multiple comparisons. In this study we used a data-driven contrast-based PLS approach with NPAIRS split-half cross-validation on an optimized PCA subspace, providing full model flexibility within the space of our neuroimaging data. Split-half resampling was chosen over bootstrapping and split-half stability, as PLS estimates using these methods may consequently result in an upward bias of the predicted correlations (Churchill et al., 2013, 2014; Kovacevic et al., 2013). We chose the PCA subspace (*k* = *11*) that simultaneously maximized prediction and reproducibility, thereby controlling for bias and adaptively removing noise variability within our dataset. Furthermore, by running the PLS analysis in an NPAIRS split-half cross-validation framework, the validity of the reported results can directly be compared to a test-retest validation study, a criterion that is necessary for strong scientific inference (Button et al., 2013; Open Science Collaboration, 2015). Our study is not without limitations. The results do not allow us to infer whether our findings reflect a 5-HTTLPR-dependent circuitry dysfunction that may have been changed or emerged during early development (Fisher et al., 2015), or whether the changes are primary or secondary to changes in brain 5-HT. To determine this, one would need to investigate a similar cohort for independent indices of 5-HT levels, such as the 5-HT_4_R agonist radioligand [11C]SB207145, that is inversely regulated in response to chronic (2–3 weeks) changes in serotonin (McMahon et al., 2016). Another limitation of this study is that our data contains samples from a previous study imposed with rather strict inclusion criteria focusing on female S' carriers. This lowers the generalizability of the results to the larger population but the longitudinal setup and the NPAIRS split-half cross-validation framework allows for strong scientific inference in this regard. In addition, our sample size is low to moderate compared to other PET studies evaluating 5-HTT fluctuations in response to a change in season, and we therefore need replication studies to confirm the results. Overall, our reported results suggest, that the seasonal 5-HTT modulations associated with the environmental stressor of winter leads to both regional up- and down-regulation of 5-HTT, and may support why the current literature on SAD points in both directions. Subject heterogeneity is important to control for, as subjects are intrinsically different in their neurobiological response to emotional changes. Strict inclusion criteria, however, come with the trade-off of being less generalizable to the general population, but may inherently contribute to the identification of reliable biomarkers within a given population. Future work may include studies, where the identified network/regions serve as an a priori hypothesis of functional coupling in a seed-based analysis using fMRI, or PET intervention studies examining the effects by which SSRIs induce clinical responses in the identified network. In conclusion, we provide novel preliminary evidence for a 5-HTT brain network that dissociates female SAD S' carriers from female S' carriers without. The identified 5-HTT brain network was comprised of a distributed set of brain regions responsive to emotion, voluntary and planned movement, executive function and memory. The findings provide additional insight into the neurobiological components through which females with the short 5-HTTLPR genotype, but with different phenotypic presentations during the environmental stressor of winter, are at risk for developing SAD and possibly predict the response to certain treatments. Furthermore, from a clinical point of view, the analysis contributes to the understanding and mapping of the complex modulation of the serotonergic system in SAD, in which the complex pathophysiology necessitates the need for individualized therapeutic strategies.

## Author contributions

GK acquired the data. MN, MG, CS, PF, NC, VB, and SS analyzed the data. MN drafted the manuscript, and MG, CS, PF, NC, VB, CG, SS, and GK revised and contributed to the final version.

### Conflict of interest statement

SS is the consulting Chief Scientific Officer at ADMdx, LLC. The other authors declare that the research was conducted in the absence of any commercial or financial relationships that could be construed as a potential conflict of interest.

## References

[B1] Avery D. H. Kizer D. Bolte M. A. Hellekson C. (2001). Bright light therapy of subsyndromal seasonal affective disorder in the workplace: morning vs. afternoon exposure. Acta Psychiat. Scand. 103, 267–274. 10.1034/j.1600-0447.2001.00078.x 11328240

[B2] Bech P. Rasmussen N. A. Olsen L. R. Noerholm V. Abildgaard W. (2001). The sensitivity and specificity of the major depression inventory, using the present state examination as the index of diagnostic validity. J. Affect. Disord. 66, 159–164. 10.1016/S0165-0327(00)00309-8 11578668

[B3] Beliveau V. Svarer C. Frokjaer V. G. Knudsen G. M. Greve D. N. Fisher P. M. (2015). Functional connectivity of the dorsal and median raphe nuclei at rest. Neuroimage 116, 187–195. 10.1016/j.neuroimage.2015.04.065 25963733 PMC4468016

[B4] Best J. Nijhout H. F. Reed M. (2011). Bursts and the efficacy of selective serotonin reuptake inhibitors. Pharmacopsychiatry 44(Suppl. 1), S76–S83. 10.1055/s-0031-1273697 21547871

[B5] Buchert R. Schulze O. Wilke F. Berding G. Thomasius R. Petersen K.. (2006). Is correction for age necessary in SPECT or PET of the central serotonin transporter in young, healthy adults? J. Nucl. Med. 47, 38–42. Available online at: http://jnm.snmjournals.org/content/47/1/38.full.pdf 16391185

[B6] Button K. S. Ioannidis J. P. A. Mokrysz C. Nosek B. A. Flint J. Robinson E. S. J.. (2013). Power failure: why small sample size undermines the reliability of neuroscience. Nat. Rev. Neurosci. 14, 365–376. 10.1038/nrn3475 23571845

[B7] Buysse D. J. Reynolds C. F. III. Monk T. H. Berman S. R. Kupfer D. J. (1989). The pittsburgh sleep quality index: a new instrument for psychiatric practice and research. Psychiatry Res. 28, 193–213. 10.1016/0165-1781(89)90047-4 2748771

[B8] Caspi A. Sugden K. Moffitt T. E. Taylor A. Craig I. W. Harrington H.. (2003). Influence of life stress on depression: moderation by a polymorphism in the 5-HTT gene. Science 301, 386–389. 10.1126/science.1083968 12869766

[B9] Cheng Y. S. Chen K. C. Yang Y. K. Chen P. S. Yeh T. L. Lee I. H.. (2011). No seasonal variation in human midbrain serotonin transporter availability in Taiwan. Psychiatry Res. 194, 396–399. 10.1016/j.pscychresns.2011.04.005 22041533

[B10] Churchill N. Afshin-Pour B. Strother S. C. (2014). PLS and functional neuroimaging: bias and detection power across different resampling schemes, in The Multiple Facets of Partial Least Squares Methods. PLS, Paris, France. Springer, Proceedings in Mathematics & Statistics, Vol.173, eds Abdi H. Vinzi V. E. Russolillo G. Saporta G. Trinchera L. (New York, NY: Springer Verlag), 93–102.

[B11] Churchill N. Spring R. Abdi H. Kovacevic N. McIntosh R. Strother S. C. (2013). The stability of behavioural PLS results in Ill-posed neuroimaging problems, in New Perspectives in Partial Least Squares and Related Methods. Springer Proceedings in Mathematics & Statistics, Vol. 56, eds Abdi H. Chin W. Esposito Vinzi V. Russolillo G. Trinchera L. (New York, NY: Springer Verlag), 171–183.

[B12] Erritzoe D. Frokjaer V. G. Haahr M. T. Kalbitzer J. Svarer C. Holst K. K.. (2010). Cerebral serotonin transporter binding is inversely related to body mass index. Neuroimage 52, 284–289. 10.1016/j.neuroimage.2010.03.086 20382236

[B13] Fischl B. (2012). FreeSurfer. Neuroimage 62, 774–781. 10.1016/j.neuroimage.2012.01.021 22248573 PMC3685476

[B14] Fisher P. M. Hariri A. R. (2012). Linking variability in brain chemistry and circuit function through multimodal human neuroimaging. Genes. Brain Behav. 11, 633–642. 10.1111/j.1601-183X.2012.00786.x 22443230

[B15] Fisher P. M. Grady C. L. Madsen M. K. Strother S. C. Knudsen G. M. (2015). 5-HTTLPR differentially predicts brain network responses to emotional faces. Hum. Brain Mapp. 36, 2842–2851. 10.1002/hbm.22811 25929825 PMC6869322

[B16] Fisher P. M. Madsen M. K. McMahon B. Holst K. Andersen S. B. Laursen H. R.. (2014). Three-week bright-light intervention has dose-related effects on threat-related corticolimbic reactivity and functional coupling. Biol. Psychiatry 76, 332–339. 10.1016/j.biopsych.2013.11.031 24439303

[B17] Grady C. L. Siebner H. R. Hornboll B. Macoveanu J. Paulson O. B. Knudsen G. M. (2013). Acute pharmacologically induced shifts in serotonin availability abolish emotion-selective responses to negative face emotions in distinct brain networks. Eur. Neuropsychopharmacol. 23, 268–278. 10.1016/j.euroneuro.2012.06.003 22739125

[B18] Greenberg B. D. Li Q. Lucas F. R. Hu S. Sirota L. A. Benjamin J.. (2000). Association between the serotonin transporter promoter polymorphism and personality traits in a primarily female population sample. Am. J. Med. Genet. 96, 202–206. 10.1002/(SICI)1096-8628(20000403)96:2<202::AID-AJMG16>3.0.CO;2-J 10893498

[B19] Greve D. N. Fischl B. (2009). Accurate and robust brain image alignment using boundary-based registration. Neuroimage 48, 63–72. 10.1016/j.neuroimage.2009.06.060 19573611 PMC2733527

[B20] Greve D. N. Svarer C. Fisher P. M. Feng L. Hansen A. E. Baare W.. (2014). Cortical surface–based analysis reduces bias and variance in kinetic modeling of brain PET data. Neuroimage 92, 225–236. 10.1016/j.neuroimage.2013.12.021 24361666 PMC4008670

[B21] Gryglewski G. Lanzenberger R. Kranz G. S. Cumming P. (2014). Meta-analysis of molecular imaging of serotonin transporters in major depression. J. Cereb. Blood Flow Metab. 34, 1096–1103. 10.1038/jcbfm.2014.82 24802331 PMC4083395

[B22] Hahn A. Haeusler D. Kraus C. Höflich A. S. Kranz G. S. Baldinger P.. (2014). Attenuated serotonin transporter association between dorsal raphe and ventral striatum in major depression. Hum. Brain Mapp. 35, 3857–3866. 10.1002/hbm.22442 24443158 PMC6868983

[B23] Hariri A. R. Goldberg T. E. Mattay V. S. Kolachana B. S. Callicott J. H. Egan M. F.. (2003). Brain-derived neurotrophic factor val66met polymorphism affects human memory-related hippocampal activity and predicts memory performance. J. Neurosci. 23, 6690–6694. Available online at: http://www.jneurosci.org/content/jneuro/23/17/6690.full.pdf 12890761 10.1523/JNEUROSCI.23-17-06690.2003 PMC6740735

[B24] Hecht D. (2010). Depression and the hyperactive right-hemisphere. Neurosci. Res. 68, 77–87. 10.1016/j.neures.2010.06.013 20603163

[B25] Holmes A. (2008). Genetic variation in cortico-amygdala serotonin function and risk for stress-related disease. Neurosci. Biobehav. Rev. 32, 1293–1314. 10.1016/j.neubiorev.2008.03.006 18439676 PMC2561331

[B26] Horschitz S. Hummerich R. Schloss P. (2001). Structure, function and regulation of the 5-hydroxytryptamine (serotonin) transporter. Biochem. Soc. Trans. 29, 728–732. 10.1042/bst0290728 11709064

[B27] Johansson C. Smedh C. Partonen T. Ekhold J. Licthermann D. Paunio T. (1999). Seasonal affective disorder and serotonin receptor polymorphisms. Mol. Psychiatry 4, S90–S91. 10.1006/nbdi.2000.0373 11300730

[B28] Johansson C. Willeit M. Levitan R. Partonen T. Smedh C. del Favero J.. (2003). The serotonin transporter promotor repeat length polymorphism, seasonal affective disorder and seasonality. Phychol. Med. 33, 785–792. 10.1017/S0033291703007372 12877393

[B29] Jovicich J. Czanner S. Greve D. N. Haley E. van der Kouwe A. Gollub R. Kennedy D.. (2006). Reliability in multi–site structural mri studies: effects of gradient non-linearity correction on phantom and human data. Neuroimage 30, 436–443. 10.1016/j.neuroimage.2005.09.046 16300968

[B30] Kalbitzer J. Erritzoe D. Holst K. K. Nielsen F. A. Marner L. Lehel S. (2010). Seasonal changes in brain serotonin transporter binding in short serotonin transporter linked polymorphic region-allele carriers but not in long-allele homozygotes. Biol. Psychiatry 67, 1033–1039. 10.1016/j.biopsych.2009.11.027 20110086

[B31] Kalbitzer J. Frokjaer V. G. Erritzoe D. Svarer C. Cumming P. Nielsen F. A.. (2009). The personality trait openness is related to cerebral 5-HTT levels. Neuroimage 45, 280–285. 10.1016/j.neuroimage.2008.12.001 19135154

[B32] Keller S. H. Svarer C. Sibomana M. (2013). Attenuation correction for the HRRT PET-scanner using transmission scatter correction and total variation regularization. IEEE Trans. Med. Imaging 32, 1611–1621. 10.1109/TMI.2013.2261313 23661313

[B33] Kendler K. Kuhn J. Vittum J. Prescott C. Riley B. (2005). The interaction of stressful life events and a serotonin transporter polymorphism in the prediction of episodes of major depression: a replication. Arch. Gen. Psychiatry 62, 529–535. 10.1001/archpsyc.62.5.529 15867106

[B34] Kim J. S. Ichise M. Sangare J. Innis R. B. (2006). PET imaging of serotonin transporters with [C-11]DASB: test-retest reproducibility using a multilinear reference tissue parametric imaging method. J. Nuclear Med. 47, 208–214. Available online at: http://jnm.snmjournals.org/content/47/2/208.full.pdf+html 16455625

[B35] King A. (2016). Neurobiology: rise of resilience. Nature 531, S18–S19. 10.1038/531S18a 26934522

[B36] Koskela A. Kauppinen T. Keski-Rahkonen A. Sihvola E. Kaprio J. Rissanen A. (2008). Brain serotonin transporter binding of [123I]ADAM: within-subject variation between summer and winter data. Chronobiol. Int. 25, 657–665. 10.1080/07420520802380000 18780196

[B37] Kovacevic N. Abdi H. Beaton D. McIntosh A. R. (2013). Revisiting PLS resampling: comparing significance vs. reliability across range of simulation, in New Perspectives in Partial Least Squares and Related Methods, Vol. 56, eds Abdi H. Chin W. Esposito Vinzi V. Russolillo G. Trinchera L. (New York, NY: Springer-Verlag), 171–183.

[B38] Krishnan A. Williams L. J. McIntosh A. R. Abdi H. (2011). Partial Least Squares (PLS) methods for neuroimaging: a tutorial and review. Neuroimage 56, 455–475. 10.1016/j.neuroimage.2010.07.034 20656037

[B39] Lam R. Levitan R. (2000). Pathophysiology of seasonal affective disorder: a review. J. Psychiatry Neurosci. 25, 469–489. 11109298 PMC1408021

[B40] Lang U. E. Hellweg R. Snader T. Gallinat J. (2009). The Met allele of the BDNF Val66Met polymorphism is associated with increased BDNF serum concentrations. Mol. Psychiatry 14, 120–122. 10.1038/mp.2008.80 19156154

[B41] Lanzenberg R. Kranz G. S. Haeusler D. Akimova E. Savli M. Hahn A. (2012). Prediction of SSRI treatment response in major depression based on serotonin transporter interplay between median raphe nucleus and projection areas. Neuroimage 63, 874–881. 10.1016/j.neuroimage.2012.07.023 22828162

[B42] Lesch K. Bengel D. Heils A. Sabol S. Z. Greenberg B. D. Petri S.. (1996). Association of anxiety-related traits with a polymorphism in the serotonin transporter gene regulatory region. Science 274, 1527–1521. 10.1126/science.274.5292.1527 8929413

[B43] MacQueen G. Frodl T. (2011). The hippocampus in major depression: evidence for the convergence of the bench and bedside in psychiatric research? Mol. Psychiatry 16, 252–264. 10.1038/mp.2010.80 20661246

[B44] Matheson G. J. Schain M. Almeida R. Lundberg J. Cselenyi Z. Borg J.. (2015). Diurnal and seasonal variation of the brain serotonin system in healthy male subjects. Neuroimage 112, 225–231. 10.1016/j.neuroimage.2015.03.007 25772667

[B45] McIntosh A. R. Bookstein F. L. Haxby J. V. Grady C. L. (1996). Spatial pattern analysis of functional brain images using partial least squares. Neuroimage 3, 143–157. 10.1006/nimg.1996.0016 9345485

[B46] McMahon B. Andersen S. B. Madsen M. K. Hjordt L. V. Hageman I. Dam H. (2016). Seasonal difference in brain serotonin transporter binding predicts symptom severity in patients with seasonal affective disorder. Brain 139, 1605–1614. 10.1093/brain/aww043 26994750

[B47] Melrose S. (2015). Seasonal affective disorder: an overview of assessment and treatment approaches. Depress. Res. Treat. 2015:178564. 10.1155/2015/178564 26688752 PMC4673349

[B48] Michalak E. E. Murray G. Levitt A. J. Levitan R. D. Enns M. W. Morehouse R.. (2007). Quality of life as an outcome indicator in patients with seasonal affective disorder: results from the can-sad study. Psyhol. Med. 37, 727–736. 10.1017/S0033291706009378 17112403

[B49] Milak M. S. Ogden R. T. Vinocur D. N. Van Heertum R. L. Cooper T. B. Mann J. J. (2005). Effects of tryptophan depletion on the binding of [11C]-DASB to the serotonin transporter in baboons: response to acute serotonin deficiency. Biol. Psychiatry 57, 102–106. 10.1016/j.biopsych.2004.09.026 15607307

[B50] Muller C. Jacobs B. (2010). Handbook of Behavioral Neurobiology of Serotonin. London: Academic Press.

[B51] Murthy N. V. Selvaraj S. Cowen P. J. Bhagwagar Z. Riedel W. J. Peers P.. (2010). Serotonin transporter polymorphisms (SLC6A4 insertion/deletion and rs25531) do not affect the availability of 5-HTT to [11C] DASB binding in the living human brain. Neuroimage 52, 50–54. 10.1016/j.neuroimage.2010.04.032 20406689

[B52] Nakajima S. Uchida H. Suzuki T. Watanabe K. Hirano J. Yagihashi T.. (2011). Is switching antidepressants following early nonresponse more beneficial in acute-phase treatment of depression?: a randomized open-label trial. Prog. Neuropsychopharmacol. Biol. Psychiatry 35, 1983–1989. 10.1016/j.pnpbp.2011.08.008 21889560

[B53] Neumeister A. Pirker W. Willeit M. Praschak-Rieder N. Asenbaum S. Brucke T. (2000). Seasonal variation of availability of serotonin transporter binding sites in healthy female subjects as measured by [123I]-2 beta-carbomethoxy-3 beta-(4-iodophenyl)tropane and single photon emission computed tomography. Biol. Psychiatry 47, 158–160. 10.1016/S0006-3223(99)00241-3 10664833

[B54] Ochsner K. N. Bunge S. A. Gross J. J. Gabrieli J. D. (2002). Rethinking feelings: an fMRI study of the cognitive regulation of emotion. J. Cogn. Neurosci. 14, 1215–1229. 10.1162/089892902760807212 12495527

[B55] Olesen O. V. Sibomana M. Keller S. H. Andersen F. L. Jensen J. Holm S. (2009). Spatial resolution of the HRRT PET scanner using 3D-OSEM PSF reconstruction, in IEEE Nuclear Science Symposium Conference Record (Orlando, FL), 3789–3790.

[B56] Open Science Collaboration (2015). Estimating the reproducibility of psychological science. Science 349:aac4716 10.1126/science.aac4716 26315443

[B57] Praschak-Rieder N. Willeit M. Wilson A. A. Houle S. Meyer J. H. (2008). Seasonal variation in human brain serotonin transporter binding. Arch. Gen. Psychiatry 65, 1072–1078. 10.1001/archpsyc.65.9.1072 18762593

[B58] Rosental N. Sack D. Gillin J. Lewy G. F. Davenport Y. Mueller P. (1984). Seasonal affective-disorder–a description of the syndrome and preliminary findings with ligh therapy. Arch. Gen. Psychiatry 41, 72–80. 10.1001/archpsyc.1984.01790120076010 6581756

[B59] Spies M. Knudsen G. M. Lanzenberg R. Kasper S. (2015). The serotonin transporter in psychiatric disorders: insights from PET imaging. Lancet Psychiatry 2, 743–755. 10.1016/S2215-0366(15)00232-1 26249305

[B60] Sureau F. C. Reader A. J. Comtat C. Leroy C. Ribeiro M. J. Buvat I.. (2008). Impact of image-space resolution modeling for studies with the high-resolution research tomograph. J. Nucl. Med. 49, 1000–1008. 10.2967/jnumed.107.045351 18511844

[B61] Tyrer A. E. Levitan R. D. Houle S. Wilson A. A. Nobrega J. N. Meyer J. H. (2016). Increased seasonal variations in transporter binding in seasonal affective disorder. Neuropsychopharmacology 41, 2447–2454. 10.1038/npp.2016.54 27087270 PMC4987850

[B62] Varnas K. Halldin C. Hall H. (2004). Autoradiographic distribution of serotonin transporters and receptor subtypes in human brain. Hum. Brain Mapp. 22, 246–260. 10.1002/hbm.20035 15195291 PMC6872082

[B63] Vincent J. L. Kahn I. Snyder A. Z. Raichle M. E. Buckner R. L. (2008). Evidence for a frontoparietal control system revealed by intrinsic functional connectivity. J. Neurophysiol. 100, 3328–3342. 10.1152/jn.90355.2008 18799601 PMC2604839

[B64] Wang L. Ashley-Koch A. Steffens D. C. Krishnan K. R. R. Taylor W. D. (2012). Impact of BDNF Val66Met and 5-HTTLPR polymorphism variants on neural substrates related to sadness and executive function. Genes. Brain Behav. 11, 352–359. 10.1111/j.1601-183X.2012.00764.x 22225729 PMC3654542

[B65] Westrin A. Lam R. W. (2007). Long-term and preventative treatment for seasonal affective disorder. CNS Drugs 21, 901–909. 10.2165/00023210-200721110-00003 17927295

[B66] Yeh Y. W. Ho P. S. Kuo S. C. Chen C. Y. Liang C. S. Yen C. H. (2015). Disproportionate reduction of serotonin transporter may predict the response and adherence to antidepressants in patients with major depressive disorder: a positron emission tomography study with 4-[18F]-ADAM. Int. J. Neuropsychopharmacol. 18, 1–12. 10.1093/ijnp/pyu120 PMC4540099 25568284

[B67] Zöllei L. Stevens A. Huber K. Kakunoori S. Fischl B. (2010). Improved tractography alignment using combined volumetric and surface registration. Neuroimage 51, 206–213. 10.1016/j.neuroimage.2010.01.101 20153833 PMC2847021

