# Supplementary material for: Brain Networks Implicated in Seasonal Affective Disorder: A Neuroimaging PET Study of the Serotonin Transporter
Source: Front Neurosci. 2017 Nov 3;11:614. doi: 10.3389/fnins.2017.00614 (PMC5682039; doi:10.3389/fnins.2017.00614)
Supplement: Supplementary file 1 [file DataSheet1.ZIP › supplementary_files/PLS_suppl_resubmission.docx]

**Brain Networks Implicated in Seasonal Affective Disorder: A Neuroimaging PET Study of the Serotonin Transporter**

Martin Nørgaard^1,2*^, Melanie Ganz^1^, Claus Svarer^1^, Patrick M. Fisher^1^, Nathan W. Churchill^4^, Vincent Beliveau^1,2^, Cheryl Grady^3^, Stephen C. Strother^3^, Gitte M. Knudsen^1,2^

^1^ Neurobiology Research Unit, Copenhagen University Hospital Rigshospitalet, Copenhagen, Denmark

^2^ University of Copenhagen, Faculty of Health Sciences, Copenhagen, Denmark

^3^ Rotman Research Institute at Baycrest, University of Toronto, Toronto, Canada

^4^ St. Michael’s Hospital, Toronto, Canada

**Supplemental material**


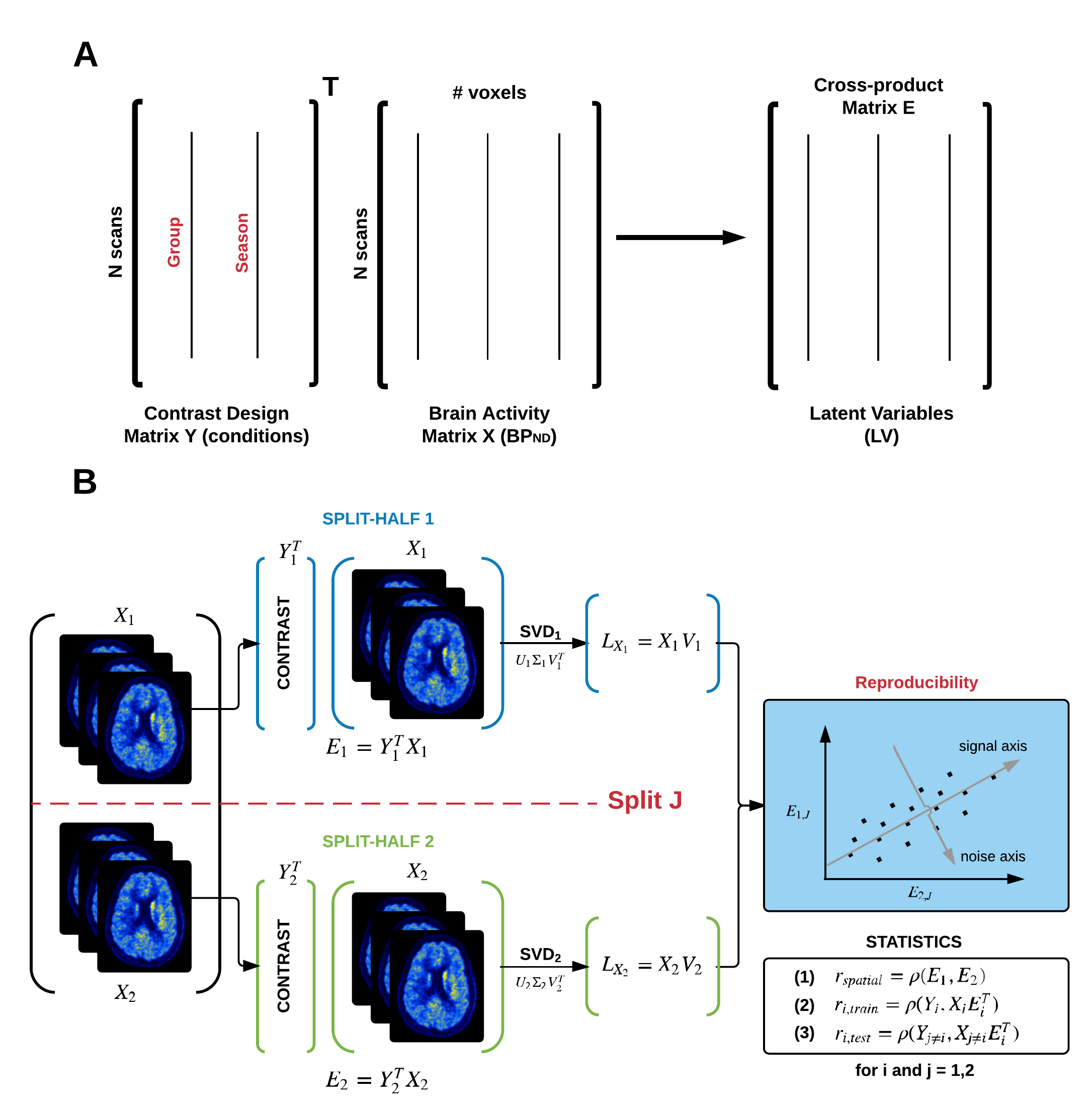


Figure S1: Principle behind NPAIRS split-half cross-validation in PLS. The data is split into a training-set and a test-set, so unbiased estimates of spatial brain patterns and brain-contrast correlation can be obtained. The procedure was executed using 1,000 splits, to stabilize the results. L is the brain score.

**Partial Least Squares on an optimized PCA basis**

In standard PLS, the design matrix **Y** is projected onto **X** in order to explain the latent basis vector **E** = **Y**T**X**. However, by initially applying a PCA to **X**, we can regularize the data space (de-noise), subsequently followed by the PLS analysis on an optimized PCA subspace. The SVD decomposes **X** into **X** = **U∆V**T. By projecting **X** onto the subset of 1 to k basis vectors,
**V**k = [v1, v2, ..., vk], we can represent **X** in an optimized k-dimensional PCA subspace (k ≤ N), yielding the matrix of basis vectors **Q**(k) = **XV**(k). Next, the PLS analysis can be executed on **Q**(k) instead of **X**, by initially centering and normalizing each PC-basis in **Q**(k), and subsequently estimating the cross- product between **Y** and **Q**(k). This can in mathematical terms be written as

**W**i = **Y**iT**Q**i (S1.1)

where **W**i expresses the projected brain pattern LV (previously **E**i) explaining the most variance in the optimized PC subspace. By projecting the test data onto the training PC-space, and subsequently onto **W**i, we estimate the predicted correlation as,

ri,test = ρ(**Y**j̸=i, **W**i(**X**j̸=i**V**i)) (S1.2)

Singular images (SI) can be obtained by projecting **V**_i_(k) back into the voxel-space, i.e. **E**i = **W**i**V**_i_(k), to estimate rtest, and additionally estimate the reproducibility, rspatial.

For further information we refer the reader to

Churchill N, Spring R, Abdi H, Kovacevic N, McIntosh R, Strother SC. The Stability of Behavioural PLS Results in Ill-Posed Neuroimaging Problems. In Abdi, H., Chin, W., Esposito Vinzi, V., Russolillo, G., & Trinchera, L. (Eds.), New Perspectives in Partial Least Squares and Related Methods. Springer Proceedings in Mathematics & Statistics Volume 56, pp 171-183, New York: Springer Verlag, 2013
