# Supplementary material for: Brain Networks Implicated in Seasonal Affective Disorder: A Neuroimaging PET Study of the Serotonin Transporter
Source: Front Neurosci. 2017 Nov 3;11:614. doi: 10.3389/fnins.2017.00614 (PMC5682039; doi:10.3389/fnins.2017.00614)
Supplement: Supplementary file 1 [file DataSheet1.ZIP › supplementary_files/PLS_suppl_resubmission.pdf]

Brain Networks Implicated in Seasonal Affective Disorder: A Neuroimaging PET Study of the Serotonin Transporter

Martin Nørgaard[1,2*], Melanie Ganz[1], Claus Svarer[1], Patrick M. Fisher[1], Nathan W. Churchill[4], Vincent Beliveau[1,2], Cheryl Grady[3], Stephen C. Strother[3], Gitte M. Knudsen[1,2]

[1] Neurobiology Research Unit, Copenhagen University Hospital Rigshospitalet, Copenhagen, Denmark

[2] University of Copenhagen, Faculty of Health Sciences, Copenhagen, Denmark

[3] Rotman Research Institute at Baycrest, University of Toronto, Toronto, Canada

[4] St. Michael's Hospital, Toronto, Canada

Supplemental material

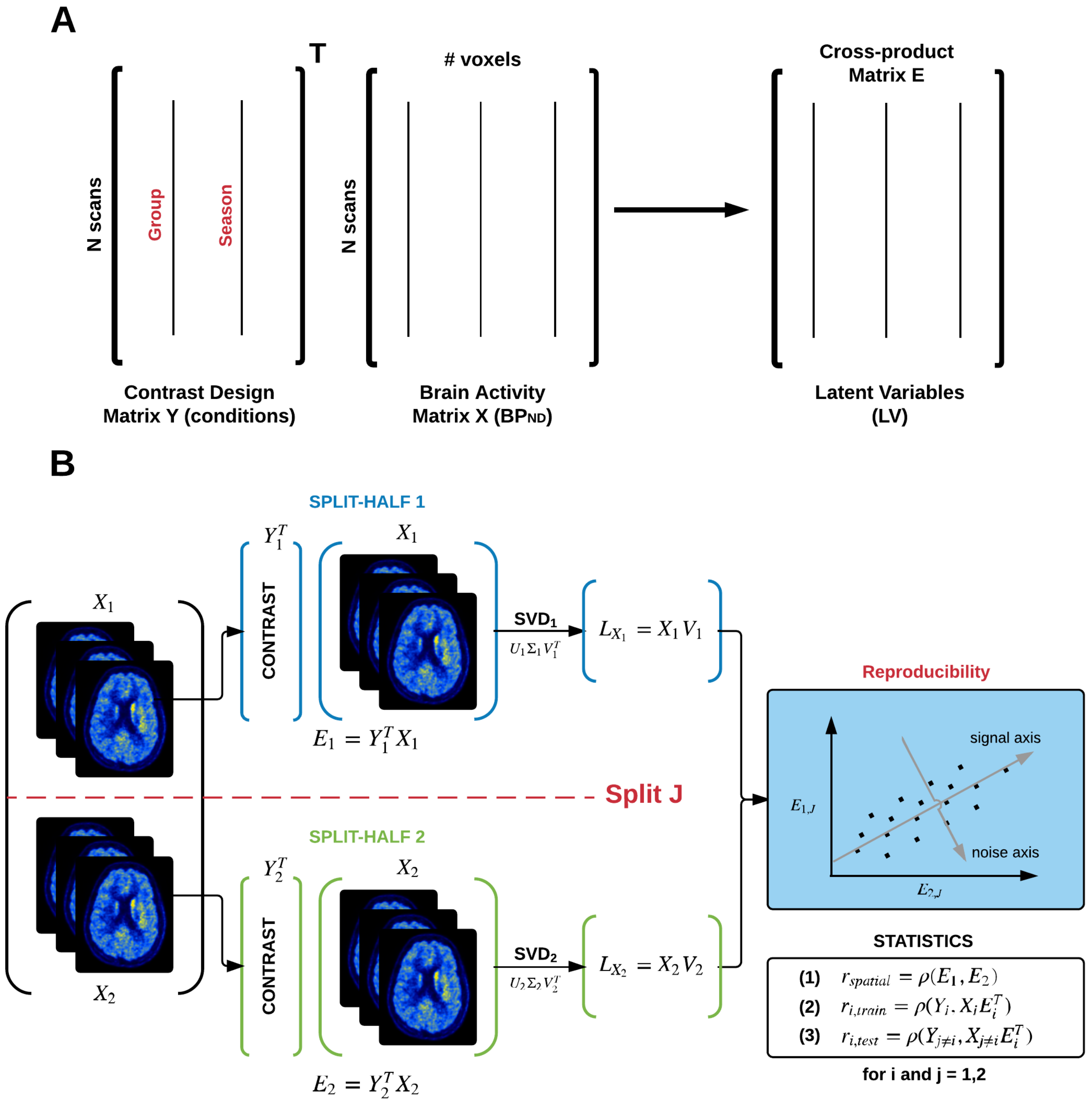

Figure S1: Principle behind NPAIRS split-half cross-validation in PLS. The data is split into a training-set and a test-set, so unbiased estimates of spatial brain patterns and brain-contrast correlation can be obtained. The procedure was executed using 1,000 splits, to stabilize the results. L is the brain score.

Partial Least Squares on an optimized PCA basis

In standard PLS, the design matrix $\mathbf{Y}$ is projected onto $\mathbf{X}$ in order to explain the latent basis vector $\mathbf{E} = \mathbf{Y}^T\mathbf{X}$. However, by initially applying a PCA to $\mathbf{X}$, we can regularize the data space (de-noise), subsequently followed by the PLS analysis on an optimized PCA subspace. The SVD decomposes $\mathbf{X}$ into $\mathbf{X} = \mathbf{U\Delta V}^T$. By projecting $\mathbf{X}$ onto the subset of 1 to k basis vectors, $\mathbf{V}^k = [v_1, v_2, ..., v_k]$, we can represent $\mathbf{X}$ in an optimized k-dimensional PCA subspace ($k \leq N$), yielding the matrix of basis vectors $\mathbf{Q}^{(k)} = \mathbf{XV}^{(k)}$. Next, the PLS analysis can be executed on $\mathbf{Q}^{(k)}$ instead of $\mathbf{X}$, by initially centering and normalizing each PC-basis in $\mathbf{Q}^{(k)}$, and subsequently estimating the cross- product between $\mathbf{Y}$ and $\mathbf{Q}^{(k)}$. This can in mathematical terms be written as

$$\mathbf{W}_i = \mathbf{Y}_i^T\mathbf{Q}_i \qquad \text{(S1.1)}$$

where $\mathbf{W}_i$ expresses the projected brain pattern LV (previously $\mathbf{E}_i$) explaining the most variance in the optimized PC subspace. By projecting the test data onto the training PC-space, and subsequently onto $\mathbf{W}_i$, we estimate the predicted correlation as,

$$r_{i,test} = \varrho(\mathbf{Y}_{j/=i}, \mathbf{W}_i(\mathbf{X}_{j/=i}\mathbf{V}_i)) \qquad \text{(S1.2)}$$

Singular images (SI) can be obtained by projecting $\mathbf{V}_i^{(k)}$ back into the voxel-space, i.e. $\mathbf{E}_i = \mathbf{W}_i\mathbf{V}_i^{(k)}$, to estimate r_{test}, and additionally estimate the reproducibility, $r_{spatial}$.

For further information we refer the reader to

Churchill N, Spring R, Abdi H, Kovacevic N, McIntosh R, Strother SC. The Stability of Behavioural PLS Results in Ill-Posed Neuroimaging Problems. In Abdi, H., Chin, W., Esposito Vinzi, V., Russolillo, G., & Trinchera, L. (Eds.), New Perspectives in Partial Least Squares and Related Methods. Springer Proceedings in Mathematics & Statistics Volume 56, pp 171-183, New York: Springer Verlag, 2013
