# Supplementary material for: Brain Networks Implicated in Seasonal Affective Disorder: A Neuroimaging PET Study of the Serotonin Transporter
Source: Front Neurosci. 2017 Nov 3;11:614. doi: 10.3389/fnins.2017.00614 (PMC5682039; doi:10.3389/fnins.2017.00614)
Supplement: Supplementary file 1 [file DataSheet1.ZIP › supplementary_files/recruitment.pdf]

Seasonal difference in brain serotonin transporter binding predicts symptom severity in patients with seasonal affective disorder

Cimbi — Center for integrated molecular brain imaging

Brenda Mc Mahon[1,2], Sofie Bech Andersen[1,2], Martin Korsbak Madsen[1,2], Liv V. Hjordt[1,2] Ida Hageman[3], Henrik Dam[3], Claus Svarer[1,2], Sophie da Cunha-Bang[1,2], William Baaré[5], Jacob Madsen[4], Lis Hasholt[6], Vibe Frokjaer[1,2] and Gitte Moos Knudsen[1,2]

[1]Neurobiology Research Unit, Rigshospitalet and University of Copenhagen, Blegdamsvej 9, 2100 Copenhagen, Denmark; [2]Center for Integrated Molecular Brain Imaging, Copenhagen, Denmark; [3] Psychiatric Centre, Copenhagen University Hospital, Rigshospitalet, Blegdamsvej 9, 2100 Copenhagen, Denmark; [4]PET and Cyclotron Unit, Rigshospitalet, Blegdamsvej 9, 2100 Copenhagen, Denmark; [5]Danish Research Centre for Magnetic Resonance, Centre for Functional and Diagnostic Imaging and Research, Copenhagen University Hospital Hvidovre, Kettegårds allé 30, 2650 Hvidovre, Denmark; [6]Department of Cellular and Molecular Medicine, University of Copenhagen, Blegdamsvej 3B, 2100 Copenhagen, Denmark

Aim

A total of 126 ^{11}C DASB PET scans were conducted to investigate if 1) absolute levels of brain SERT binding differs between patients with SAD and healthy controls (HC), 2) if seasonal SERT fluctuations differ between groups and 3) if development of depressive symptoms is predicted by SERT changes. Seventeen SAD patients and 23 HC were assed biannually allowing us to do the first longitudinal investigation of SAD patients.

Introduction

It is estimated that more than 15% of Copenhagen inhabitants suffer from SAD or sub-syndromal SAD [1]. Cross-sectional neuroimaging studies have demonstrated that healthy carriers of the short 5-HTTLPR polymorphism (S-carriers) have higher serotonin transporter (SERT) binding in the winter than the summer.

Methods and Results

A measure of global cerebral SERT binding (global BP_{ND}) was calculated from a volume weighted average of 17 grey matter segmented SERT regions (subcortical and cortical raphe projection areas).

Group differences in absolute levels of global BP_{ND} between SAD and HC were compared summer and winter. SAD patients have significantly lower SERT levels than HC in the summer but there were no group differences in the winter.

In the longitudinal study, we used the relative change in SERT from winter to summer ($relBP_{ND}$) as outcome variable. We found a highly significant group effect (p=0.0002); SAD patients had higher SERT in the winter compared to summer whereas the opposite was seen in the HC (table 1 & fig.3).

In SAD patients, we tested if $relBP_{ND}$ predicted the relative change in symptom scores from winter to summer (relSIGH-SAD) and found that a larger seasonal difference was associated with the largest difference in SAD symptoms (table 1 & fig.4).

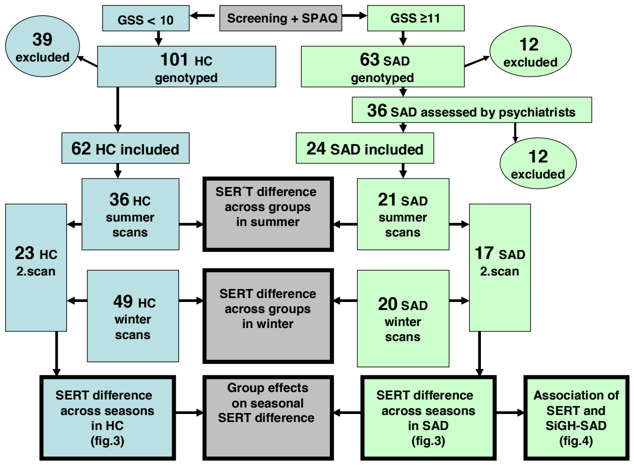

Inclusion and data analysis. SAD: Seasonal affective disorder patients, HC: Healthy controls, SPAQ: The seasonal pattern assessment questionnaire, GSS: Global seasonality score, SIGH-SAD: Hamilton depression rating scale, SAD version.

	Summer (mean±SD)	Winter (mean±SD)	*P-value*
**Healthy controls, n=23**			
Global BP_{ND}	0.49±0.08	0.46±0.06	0.01
MDI score	5.4±3.6	5.0±3.5	0.49
PSQI global score	3.7±2.1	3.6±1.8	0.79
**SAD, n=11 (S-carriers)**			
Global BP_{ND}	0.45±0.07	0.51±0.05	0.035
SIGH-SAD score	2.27±2.61	20.3±8.3	<0.0001
**SAD, n=17 (total)**			
Global BP_{ND}	0.47±0.07	0.50±0.06	0.065
MDI score	6.4±4.2	21.4±7.9	P<0.0001
PSQI global score	4.5±1.8	6.5±2.3	0.016

Table 1: Summer and winter PET-data and psychometrics. PSQI: Pittsburgh Sleep Quality Index, MDI: Major Depression Inventory (MDI), two tailed p-values from paired t-tests.

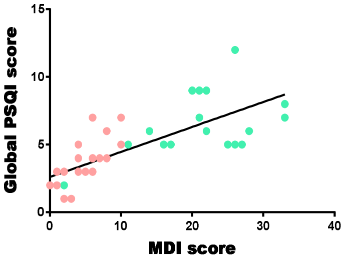

Figure 1: Mood and sleep quality correlated both summer (not shown) and winter for healthy individuals (pink dots) and SAD patients (mint dots), R^2=0.54, β=0.19, p< 0.0001.

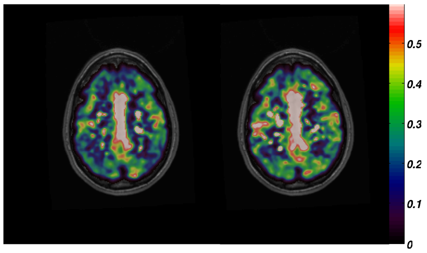

Figure 2: A ^{11}C DASB PET images illustrating the SERT levels of a typical SAD patient, a young S-carrying female. In the winter (right-hand side) the SERT levels are 32% higher compared to summer (left-hand side).

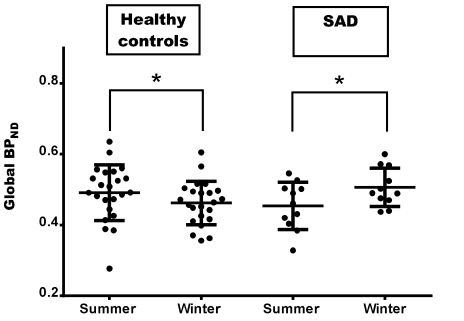

Figure 3: S-carriers diagnosed with SAD (n=11) showed a significant up-regulation in global SERT in the winter compared to summer (p=0.04). Healthy S-carriers (n=23) had a significant down-regulation in global SERT in the winter (p=0.01).

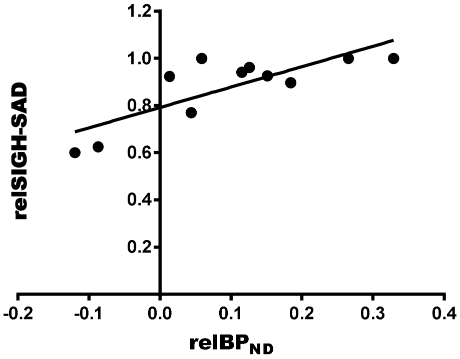

Figure 4: Relative difference in symptom severity (SIGH-SAD scores winter-summer difference relative to winter (relSIGH-SAD)) versus relative difference in global cerebral SERT binding (BP_{ND} winter-summer difference relative to winter ($relBP_{ND}$)) in 11 SAD S-carriers (n=11): β=0.87, R^2=0.65, p=0.003.

Conclusions

Healthy individuals down-regulate their SERT in winter compared to summer and SAD patients up-regulate their cerebral SERT to an extent that correlates with their SAD symptom severity. Our observations suggest a failure in SAD to regulate cerebral SERT appropriately during exposure to the environmental stress of winter, thereby contributing to the development of SAD symptoms.

References

[1] Dam H., Jakobsen k., Melleruo E., 1998. Prevalence of winter depression in Denmark. Acta Psychiatr Scand 97, 1-4.

Acknowledgements

The Lundbeck Foundation, University of Copenhagen and The John & Birthe Meyer Foundation are acknowledged for financial support. Lone Freyr, Bente Dall and Agnete Dyssegaard are greatly appreciated for technical assistance.

Contact: Brenda@nru.dk

P.1.i.037

Patients with seasonal affective disorder show seasonal fluctuations in their cerebral serotonin transporter binding

B. Mc Mahon[1], S.B. Andersen[1], M.K. Madsen[1], L.V. Hjordt[1], I. Hageman[2], H. Dam[2], C. Svarer[1], S. Da Cunha-Bang[1], W. Barré[3], J. Madsen[4], L. Hasholt[5], V. Frokjaer[1], G.M. Knudsen[1]

[1]Copenhagen University Hospital Rigshospitalet, Neurobiology Research Unit, Copenhagen, Denmark

[2]Copenhagen University Hospital Rigshospitalet, Psychiatric Centre, Copenhagen, Denmark

[3]Copenhagen University Hospital Hvidovre,

Danish Research Centre for Magnetic ResonanceCentre for Functional and Diagnostic Imaging and Research, Copenhagen, Denmark

[4]Copenhagen University Hospital Rigshospitalet, PET and Cyclotron Unit, Copenhagen, Denmark

[5]University of Copenhagen, Department of Cellular and Molecular Medicine, Copenhagen, Denmark

Objectives: Lack of daylight is a prominent environmental stressor at high latitudes. It is estimated that more than 15% of the Copenhagen inhabitants suffer from Seasonal Affective Disorder (SAD) or sub-syndromal SAD [1]. Cross-sectional neuroimaging studies have demonstrated that in healthy individuals, striatal serotonin transporter (SERT) binding is high at winter solstice and low at summer solstice. These fluctuations are particularly evident in carriers of the short 5-HTTLPR polymorphism (S-allele carriers). The aim of the present study is to do the first longitudinal investigation of seasonal SERT fluctuations in healthy S-allele carriers and in S-allele carriers suffering from SAD.

Methods: All participantscompleted the Seasonal Pattern Assessment Questionnaire (SPAQ) to evaluate seasonal variations in sleep, social activity, mood, weight, appetite and energy. The score on each item was summed to obtain a Global Seasonality Score (GSS), which indexes the degree of winter depression (range: 0–24, GSS>10 indicates SAD). Subjects without any seasonality and a GSS equal to or less than 10 entered the study as healthy participants while subject with a GSS score equal to or higher than 11 were interviewed by specialized psychiatrists both in their asymptomatic and their symptomatic (winter) phase to establish the SAD diagnosis. Twenty-three (13 females) healthy S-allele carriers (mean±SD: GSS: 4.8±2 and age: 25±7 years) and 11 (six females) S-allele carrying SAD patients (mean±SD: GSS:13.7±2 and age: 26±8 years) were investigated with a dynamic [^{11}C]DASB HRRT PET scan and a MRI brain scan both summer and winter, in randomized order. Non-displaceable binding potential (BP_{ND}) was quantified using MRTM2. Summer BP_{ND}s were plotted as a function of winter BP_{ND}s for 17 different brain regions [2]. The slope of the regression line (β) was used as a measure of individual change in global brain SERT changes across seasons. β values were compared between healthy controls and SAD patient using a Mann–Whitney unpaired t-test. A one sample paired t-test was used within groups to investigate significant seasonal SERT changes.

Results: We found a significant difference between healthy controls and SAD patients in seasonal SERT changes: median β healthy controls: 1.033, median β SAD: 0.93, U=59, p=0.01. Furthermore we observed a tendency for a winter-summer change in the SAD group (β≠1): t(10)=2.136, meanβ±SD: 0.96±0.07, p=0.058 but not in the healthy control group: t(22)=1.759, mean β±SD: 1.024±0.07, p=0.092.

Conclusions: We find that SAD patients experience a significantly larger seasonal SERT fluctuation compared to their healthy counterparts. We were not able to reproduce previous findings of a similar up-regulation during winter in healthy subjects and we speculate that this is due to a careful selection of individuals completely void of season related symptoms. Our data suggests that seasonally provoked depression is linked to seasonal SERT changes.

1. Dam H., Jakobsen k., Melleruo E., 1998. Prevalence of winter depression in Denmark. Acta Psychiatr Scand 97, 1–4.

2. Haahr, M.E., Fisher P.M., Jensen C.G. et al. 2014. Central 5-HT4 receptor binding as biomarker of serotonergic tonus in humans: a 11C SB207145 PET study. Mol Psychiatry 19(4), 427–32.

Disclosure statement: Research support: The Lundbeck Foundation.

Citation: Eur Neuropsychopharmacol. 2014;24(Suppl 2):S319

Keywords
Serotonin
Biological rhythms
Brain imaging
